# Epigenetic DNA Methylation Under the Influence of Low-Dose Ionizing Radiation, and Supplementation with Vitamin B12 and Folic Acid: Harmful or Beneficial for Professionals?

**DOI:** 10.3390/epigenomes9020017

**Published:** 2025-05-31

**Authors:** Borivoje Savic, Bozidar Savic, Svetlana Stanojlovic

**Affiliations:** 1 University Clinical Centre of Serbia, University Eye Hospital, Pasterova 2, 11000 Belgrade, Serbia; 2 Institute of Veterinary Medicine of Serbia, Janisa Janulisa 14, 11000 Belgrade, Serbia; 3 Faculty of Medicine, University of Belgrade, 11000 Belgrade, Serbia

**Keywords:** epigenetics, methylation, dosimetry-chromosomal aberrations

## Abstract

This review paper highlights the importance of educating current and future professionals about epigenetic mechanisms and recognizing epigenetics as a crucial model for protection against ionizing radiation. Two basic models for radiation-induced DNA damage are currently in use. The association between mutations and chromosomal aberrations provides a framework for analyzing risks at low radiation doses and exposure to small doses. However, there is no monitoring of epigenetic changes in professionals exposed to low doses of ionizing radiation. Epigenetic events regulate gene activity and expression not only during cell development and differentiation but also in response to environmental stimuli, such as ionizing radiation. Furthermore, the potential occurrence of malignant and hereditary diseases at low doses of ionizing radiation is linearly correlated and is considered a scientifically accepted assumption, despite recognized scientific limitations associated with this assessment. The aim of this review is to integrate novel and intriguing radiobiological paradigms regarding the effects of ionizing radiation on DNA methylation and epigenetic regulation of the DNA molecule. Several hypothesized biological responses to ionizing radiation are examined, linking them to epigenetic mechanisms involved in health risk assessment for professionals. The second part of the review includes published research related to epigenetics, supplementation, and virus reactivation in the context of epigenetic modifications of the DNA molecule. We hypothesize that different cycles lead to changes in the epigenome, which may be associated with the reactivation of certain viruses and the deficiency of specific dietary elements. These findings are linked to minimal deficiencies in vitamin B12 and folic acid, which may contribute to epigenomic changes. This aspect is crucial for the immune status of individuals working in high-risk environments.

## 1. Introduction

Cellular dysfunction can arise as a random biological issue. This problem is influenced by two factors: “poor” genetic inheritance and various harmful environmental factors or habits, such as stress, smoking, alcohol consumption, ionizing radiation, environmental pollution, and so on. The epigenetic regulation of viral reactivation is associated with DNA methylation [1]. Changes related to DNA methylation lead to modifications in the histone tails of cellular chromatin. These changes are an integral part of the epigenetic regulation of chromatin activity at the cellular level [2]. Biological dosimetry in individuals occupationally exposed to ionizing radiation, as well as the detection of potential chromosomal aberrations in such individuals, provides only partially accurate dosimetric data, but does not incorporate epigenetic aspects [2,3].

Many studies have provided evidence supporting the idea that the cellular response to low-dose radiation is, at least partially, regulated by gene expression networks. A review of the literature indicates that epigenetic mechanisms are involved in the adaptive response [3,4,5] and that changes in DNA methylation occur even more frequently than genetic mutations [4].

Global DNA methylation loss in certain genes was the first epigenetic abnormality identified in cancer cells [6]. The presence of epigenetic changes, particularly in the DNA methylation pattern, has been associated with ionizing radiation [6,7,8]. It is extremely difficult to precisely determine the impact of a substance or environment on the epigenome, but some modulators of DNA molecules responsible for gene expression and immune response to specific pathogens, such as ionizing radiation, have been identified [8]. The DNA molecule acts like a sponge that absorbs ionizing radiation. However, before changes occur in the DNA molecule itself, alterations can be observed in the cell’s epigenome [7,8]. Double-strand DNA breaks, the most serious DNA lesions caused by ionizing radiation, are also caused by deficiencies in several vitamins or minerals, such as folate. Several studies have shown that these DNA damage biomarkers are associated, in cross-sectional studies, with intake levels of folate and certain nutrients, including vitamins B-2, B-3, B-6, and B-12, zinc, magnesium, and manganese, which are directly linked to protection against DNA damage [9,10].

## 2. DNA Methylation and Possible Connection with Ionizing Radiation Mechanisms

**Figure 1:**
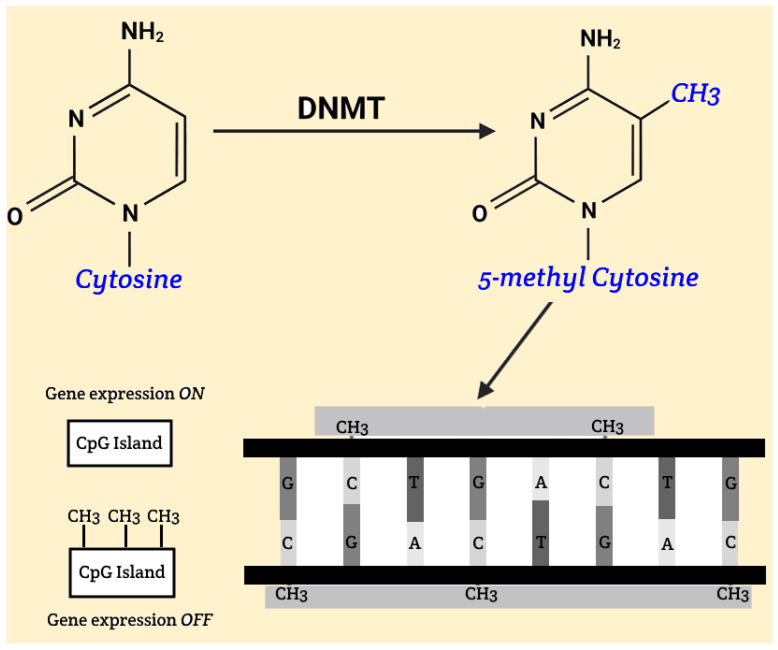
Gene silencing, DNA methylation.

The fundamental mechanism of DNA methylation involves the addition of methyl groups to cytosine bases within CpG islands, significantly affecting gene activity. CpG islands are typically regions located in promoters and pre-promoters, consisting of at least 200 base pairs, with cytosine and guanine comprising at least 50% of the sequence. Hypomethylation, which can occur under the influence of ionizing radiation, may lead to increased activity of genes that are otherwise weakly expressed or completely inactive. This can cause significant changes, ranging from harmless phenotypic variations to increased tumor cell proliferation, potentially resulting in malignant diseases. During embryonic development, methylation of CpG islands plays a crucial role in gene silencing, which is essential for normal development. With aging, there is a gradual hypomethylation of previously methylated DNA regions and hypermethylation of unmethylated CpG islands, leading to instability and altered gene activity. Malignancies are often associated with hypomethylated oncogenes and hypermethylated tumor suppressor genes [Figure 1].

**Figure 2:**
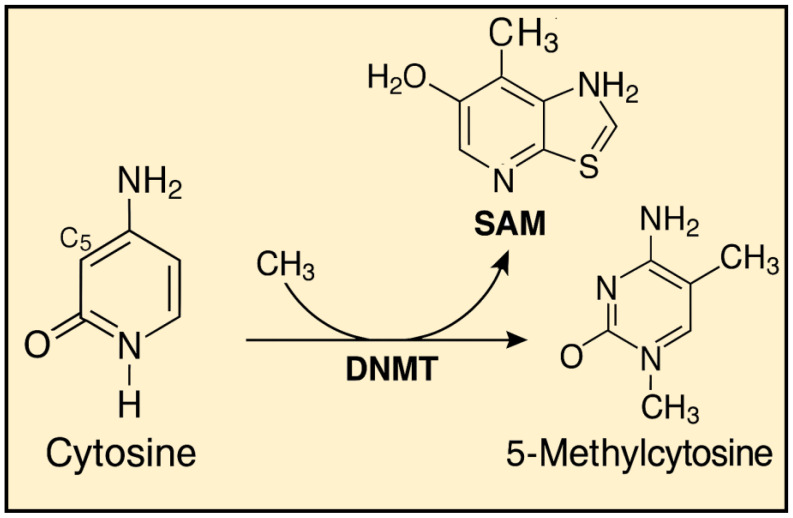
Cytosine Methylation to 5-Methylcytosine Mediated by DNMT and SAM.

These disturbances most commonly arise due to dietary and environmental factors, including the influence of ionizing radiation. Proper DNA methylation is believed to contribute to the organism’s homeostasis. Studies have shown that diets rich in nutrients and biologically active components that act as methyl group donors can have a positive impact on health. The methylation mechanism itself relies on the conversion of cytosine to 5-methylcytosine, catalyzed by enzymes known as DNA methyltransferases (DNMTs) [Figure 2]. Currently, five enzymes from this group are known: Dnmt1, Dnmt2, Dnmt3a, Dnmt3b and DnmtL [11].

Dnmt1 is the enzyme responsible for maintaining methylation patterns on hemimethylated DNA during cell division, acting on genes with an already established pattern. The role of Dnmt2 in methylation is not fully understood, while Dnmt3a, Dnmt3b, and DnmtL are involved in establishing new methylation patterns. The initial methylation pattern is set during gametogenesis and embryonic development, after which it is erased and reestablished according to the individual’s sex. In male fetuses, there is an increase in the overall genome methylation, while in female fetuses, methylation is initially reduced and established later. This methylation pattern is transmitted through all germ cells and differs from the pattern formed in somatic cells [12].

## 3. The Impact of Low Doses of Ionizing Radiation

It is certainly not a trivial question whether the stimulation of cellular defense mechanisms by low levels of radiation is beneficial or harmful in terms of health effects on the human body. Although this stimulation sometimes resembles a positive reaction, this cannot be the case if, for example, cells damaged by prolonged radiation exposure disrupt their division rhythm and avoid apoptosis, leading to genomic instability and potential disease [8,13]. Tumor promotion by increasing the survival probability of cells with accumulated damage or mutations is undoubtedly detrimental [7].

Some clinical and experimental evidence suggests that low radiation doses can shift the functional profile of CD4+ T cells toward the Th2 phenotype. Damage to CD8+ T cells tends to be less consistent, possibly due to variations in radioresistance, which may be linked to activation status [14].

The eye lens is considered one of the most sensitive tissues; biological and mechanical studies in both in vitro and in vivo models have identified several processes contributing to lens opacification and cataract development. These include DNA damage and repair mechanisms, extracellular matrix effects, membrane damage, dysregulation of protein and gene expression, oxidative damage, and intercellular communication [15,16].

Radiation-related cancer risks vary depending on the organ or tissue affected. The most sensitive tissues are bone marrow, brain, thyroid gland, skin, and breasts, although radiation exposure has been linked to cancer risks in nearly every tissue and organ in the body. Cancers that have not been consistently or convincingly associated with low radiation doses include chronic lymphocytic leukemia, Hodgkin’s and non-Hodgkin’s lymphoma, and melanoma [17,18].

The heart and vascular system have long been recognized as potential targets for the harmful effects of radiation exposure. In 2012, the International Commission on Radiological Protection (ICRP) issued a statement advising physicians to be aware of the absorbed dose threshold for cardiovascular patients [19].

Experimental findings from studies on fruit flies (Muller, 1927) and mice (Russell, 1951) demonstrated that ionizing radiation causes hereditary genetic effects [20]. However, human data have not consistently replicated these experimental findings, often due to limited statistical power from small sample sizes. Nonetheless, its impact on epigenetics has been clearly observed [20]. A recent literature review on potential cognitive impairments associated with low-dose radiation exposure concluded that both biological and epidemiological studies provide evidence of such changes, but a better characterization of effects and a deeper understanding of the underlying mechanisms are needed [20].

## 4. Transmethylation, Vitamin B12, and Folic Acid

**Figure 3:**
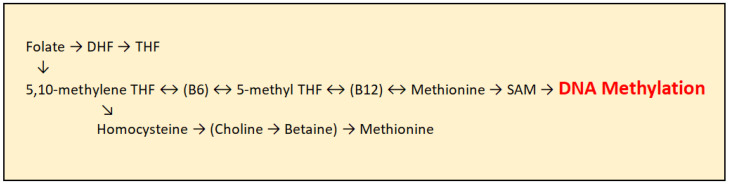
Transmethylation cycle: DHF (Dihydrofolate), THF (Tetrahydrofolate), DMG (Dimethylglycine), SAM (S-adenosylmethionine), and SAH (S-adenosylhomocysteine).

As humans cannot synthesize methyl groups independently, they must ingest them through foods rich in certain nutrients, with folic acid being the most important methyl group donor. In addition to folic acid, vitamin B12 and other B-complex vitamins play an important role. Before entering the transmethylation cycle, folate is first reduced to dihydrofolate (DHF) and then converted into tetrahydrofolate (THF). THF is subsequently transformed into 5,10-methylene-THF through the action of the enzyme serine hydroxymethyltransferase, with several B-complex vitamins acting as cofactors. Further, 5,10-methylene-THF is converted into 5-methyl-THF by the enzyme methylenetetrahydrofolate reductase (MTHFR), with vitamin B2 and FAD serving as essential cofactors. 5-methyl-THF then donates a methyl group to homocysteine, which is converted into methionine with the assistance of vitamin B12 as a cofactor. Methionine subsequently serves as a precursor for the synthesis of S-adenosylmethionine (SAM), the principal methyl group donor for DNA methylation, catalyzed by Dnmt enzymes [Figure 3]. Folic acid itself cannot directly donate methyl groups due to insufficient transfer potential. S-adenosylmethionine is the activated donor formed by transferring an adenosyl group from ATP to the sulfur atom of methionine, making the molecule highly reactive [21].

## 5. Modifications in Histone Tails

Histones are highly conserved alkaline proteins rich in identical amino acid residues that are susceptible to post-translational modifications (PTMs) on their N- and C-terminal tails. The covalent attachment or removal of molecules or functional groups to the N-terminal regions of histone tails significantly influences chromatin structure. The core histones include histone H2A, H2B, H3, and H4, along with histone H1, the linker histone. Approximately 146 to 147 base pairs of DNA are wrapped in a left-handed superhelical structure around each histone octamer, which consists of two copies of each core histone. Key histone modifications include methylation, acetylation, and phosphorylation. Chromatin structure can be altered by nucleosome remodeling, histone variant exchange, or changes in nucleosome positioning, all of which affect chromatin compaction. Post-translational histone modifications play a central role in organizing chromatin architecture, especially in maintaining the balance between euchromatin, which is transcriptionally active, and heterochromatin, which is transcriptionally silent. These modifications can lead to either gene activation or repression, depending on the type and position of the functional group and the modified amino acid residue [22,23].

Histone methylation is a post-translational modification catalyzed by histone methyltransferases (HMTs). This modification involves the transfer of a methyl group (–CH_3_), derived from S-adenosylmethionine (SAM) to lysine or arginine residues on histone proteins. Methyl groups bound to lysine or arginine residues can be removed by histone demethylases (HDMs). Lysine residues in histones can be mono-, di-, or trimethylated. Histone methylation plays a significant role in the regulation of gene expression and may lead to either gene activation or silencing, depending on the specific position of the modified amino acid residue within the protein and the degree of methylation. This complex regulation underscores the importance of context-specific factors in determining the functional outcome of histone modifications.

Acetylation is another important post-translational modification, involving the addition of an acetyl group to lysine residues on histone tails. Enzymes known as histone acetyltransferases (HATs) and histone deacetylases (HDACs) are responsible for the acetylation and deacetylation of histone tails within nucleosomes. These reversible modifications are closely associated with changes in gene expression, with HATs generally functioning as transcriptional coactivators, while HDACs act as corepressors.

Histone phosphorylation is catalyzed by a variety of kinases, including cyclin-dependent kinases and mitogen- and stress-activated protein kinases (MSKs). Most phosphorylation events occur on serine residues of histone H3 at positions 10 and 28, as well as on threonine residues at positions 3, 6, and 11. Histone phosphorylation is a significant post-translational modification that occurs on serine, threonine, and tyrosine residues in histone tails.

As mentioned, this process is catalyzed by enzymes known as histone kinases, which transfer phosphate groups from ATP to specific amino acid residues in histones. Histone phosphorylation plays a crucial role in regulating chromatin structure and gene expression, acting as a signal for various biological processes, including DNA repair, RNA synthesis, and chromatin organization [24,25].

## 6. Histone Modifications Induced by Ionizing Radiation

Histone modifications triggered by ionizing radiation represent a critical exogenous factor influencing epigenetic regulation, with significant implications for chromatin organization and gene expression. Understanding these mechanisms can provide insights into the epigenetic effects of radiation exposure and potential therapeutic strategies for mitigating radiation-induced DNA damage.

Exposure to ionizing radiation leads to various histone modifications essential for the DNA damage response. One of the most notable is the phosphorylation of histone H2AX at serine 139 (known as γ-H2AX), which serves as an early cellular response marker to DNA double-strand breaks (DSBs). This modification initiates signaling cascades that activate DNA repair pathways and help preserve genomic integrity [26].

In vivo studies on mouse models have shown that low-dose X-ray exposure reduces histone H4 trimethylation in the thymus, correlating with decreased chromatin compaction. These alterations are also associated with global DNA hypomethylation and DNA damage accumulation, accompanied by reduced expression of DNA methyltransferases (DNMTs). Similar patterns of histone methylation changes have been observed in human breast cancer samples, suggesting that radiation-induced modifications of DNA and histone methylation can severely impact genome integrity.

Furthermore, histone acetylation plays a crucial role in DNA repair processes, including chromatin remodeling, DSB marking, repair factor recruitment, cell cycle regulation, and apoptosis [6,27,28].

## 7. Epigenome, Folate Supplementation, and DNA Methylation

Epigenetic research has enabled the identification of the epigenetic status of specific genomic loci. This process, also referred to as epigenetic profiling, allows for the determination of the epigenetic effects of particular nutrients on targeted genes of interest. In this way, individuals at increased risk of developing certain diseases due to exposure to ionizing radiation can be more appropriately counseled, or at least considered for potential lifestyle interventions, such as supplementation, based on the findings obtained [29].

In DNA methylation, the most important nutrients are B-complex vitamins, especially folic acid, as well as compounds like betaine, homocysteine, and methionine, involved in the transmethylation cycle. Among these, folic acid (vitamin B9) is the most researched and significant. The parent molecule of folates, pteroylglutamic acid, consists of three parts: the 2-amino-4-hydroxypteridine core, p-aminobenzoic acid (PABA), and L-(+)-glutamic acid.

The pteridine ring within the core structure is connected to PABA (para-aminobenzoic acid) via a methylene bridge, while PABA is linked to glutamic acid through an amide bond.

However, this form of folate is not commonly found in nature and is not metabolically active. The biologically active forms are the reduced derivatives—tetrahydrofolates (THFs)—which function in the body as coenzymes for the transfer of one-carbon (C1) units. THF primarily transfers methyl groups within the transmethylation cycle but can also transfer formyl and methylene groups, which are essential for normal DNA synthesis and repair, as well as RNA synthesis. Most folate compounds in the body exist as polyglutamates, typically containing five to seven glutamate residues linked by gamma-peptide bonds.

In our diet, the majority of folates are consumed in a form bound to a chain of amino acids, predominantly glutamate, forming polyglutamates. Additionally, a smaller amount of folic acid is ingested through food; although folic acid is not biologically active, it exhibits approximately twice the bioavailability compared to natural folates. As previously mentioned, dietary folic acid must be converted into its active form within the body. This conversion occurs in the liver, where monoglutamate forms or free folic acids are transformed into dihydrofolate through the action of the enzyme dihydrofolate synthase. Subsequently, dihydrofolate is reduced to tetrahydrofolate (THF) by dihydrofolate reductase. THF is then converted into 5,10-methylene-THF, the biologically active form of folate, through the action of serine hydroxymethyltransferase [30].

Vitamin B12 is typically ingested as cyanocobalamin, mainly sourced from animal-derived proteins. Once inside the cell, B12 is activated by accepting a methyl group in the transmethylation cycle, acting as a cofactor in the remethylation of homocysteine to methionine and in the conversion of methylTHF to THF. Vitamin B12 also plays an essential role in fatty acid and amino acid metabolism, serving as a cofactor in the conversion of L-methylmalonyl-CoA to succinyl-CoA for the citric acid cycle. A deficiency in vitamin B12 can disrupt DNA synthesis and methylation, as well as fat and amino acid metabolism, often due to reduced intake or absorption problems such as hypochlorhydria [31].

## 8. Vitamin B12, Folic Acid, and Low Doses of Ionizing Radiation

Vitamin B12 and folic acid are essential for DNA methylation, a key epigenetic mechanism regulating gene expression [32]. Since humans cannot synthesize methyl groups de novo, they must be obtained through diet or supplementation [29,33,34]. Folate status, in particular, has been directly linked to DNA methylation patterns and genomic stability.

Exposure to ionizing radiation can disrupt the epigenetic landscape, with DNA demethylation being one of the characteristic changes observed. Given this, adequate intake of B vitamins—especially in occupational settings involving radiation—may mitigate some of the adverse epigenetic effects associated with such exposure [29,33].

Recent advances in epigenetic profiling allow for the assessment of locus-specific methylation changes, providing insight into how specific nutrients can modulate gene expression. Suboptimal levels of vitamin B12 and folate, whether due to dietary insufficiency or environmental factors like radiation, may negatively influence both the immune response and epigenetic regulation in exposed individuals.

Evidence suggests that supplementation with folic acid and vitamin B12 offers protective benefits for DNA exposed to ionizing radiation. Folate deficiency, for example, has been associated with uracil misincorporation into DNA, global hypomethylation, impaired DNA repair, and increased chromosomal instability—including missegregation and strand breaks [29,33,34,35]. These risks may be exacerbated by radiation, which further challenges DNA repair mechanisms and chromosomal integrity.

Beyond B vitamins, several micronutrients—such as zinc, vitamins A, C, and E, lycopene, curcumin, selenium, and proanthocyanidins—have shown promise in supporting DNA repair and reducing oxidative stress. Their combined supplementation may help preserve genomic and epigenomic integrity, particularly in high-risk occupational environments [32,33,34,35].

## 9. Epigenetics and Possible HSV-1 Reactivation in Professionals

Herpes simplex virus type 1 (HSV-1), like other alpha-herpesviruses, has the ability to establish latency within sensory ganglia. During the latent phase, the virus persists as a non-integrated episome associated with nucleosomes in the host cell nucleus. In this state, transcription of the viral genome is largely restricted to the Latency-Associated Transcript (LAT), while the lytic genes remain transcriptionally repressed. This division of the viral genome into active and inactive regions suggests that epigenetic mechanisms play a crucial role in controlling the latent expression of HSV-1 genes.

HSV-1 infection is typically acquired through primary exposure of the mucosal surfaces of the lips or eyes. During the initial acute infection, the virus replicates locally within the epithelial cells of the mucosa, then enters the sensory nerve endings beneath the skin and is transported retrogradely to the trigeminal ganglion. There, the virus either replicates in certain cells or establishes latency in others. During latency, the virus remains dormant within the trigeminal ganglion without the production of infectious particles, and viral gene expression, apart from LAT, is suppressed.

Various stressors, including exposure to ionizing radiation, can trigger periodic reactivation of the virus. Upon reactivation, lytic transcription of viral genes and viral DNA replication are initiated in some neurons, leading to the anterograde transport of newly formed virions back to the site of the primary infection. This process may result in the reappearance of infectious virus at the original site and, in some cases, the development of clinical lesions such as herpes manifestations [36].

Upon entry into the neuron, HSV-1 can either undergo lytic replication or establish a latent infection. During the acute or lytic phase, all three kinetic classes of viral genes—immediate early (IE), early (E), and late (L)—are expressed, and the viral genome is replicated through a linear mechanism. In contrast, latent infection is characterized by circularization of the viral genome and suppression of lytic gene expression. During latency, transcription is almost exclusively limited to a single non-coding gene product, the Latency-Associated Transcript (LAT), which is produced in large quantities [37].

## 10. Mechanism of Possible Epigenetic Association Between Immune Reactivation of Herpes Simplex Virus (HSV-1) and Additional Supplementation with Low Doses of Ionizing Radiation

Epigenetic regulation of viral reactivation is linked to DNA methylation. Changes in DNA methylation lead to modifications in the histone tail of cellular chromatin. These alterations are an integral part of the epigenetic regulation of viral activity at the level of cellular chromatin [38]. During the latent phase of HSV-1, the LAT (Latency-Associated Transcript) region undergoes various changes in histone arrangement [37,39].

Vitamin B12 and folic acid are involved in the process of DNA methylation, which is one of the fundamental mechanisms of epigenetic regulation of DNA molecules. DNA methylation is associated with folate intake and folate concentration levels in the body [33]. As previously mentioned, the latent viral genome contains a transcriptionally active LAT region. The activity of the LAT region is directed toward chromatin organization and does not involve known coding proteins. This type of LAT region activity suggests an epigenetic regulatory mechanism.

### 10.1. Possible Epigenetic Stress Induced by Ionizing Radiation and Reactivation of HSV-1 Virus

Epigenetic changes in the DNA molecule are crucial for the human organism, as they involve modifications related to gene expression without affecting the primary structure of the DNA molecule. Several changes contribute to the epigenetic regulation of DNA, including DNA methylation, histone modifications, and other chromatin alterations [5].

The model by which the epigenome is altered is partially hereditary and partially dependent on an individual’s lifestyle. Epigenomic changes are significantly influenced by environmental factors, including ionizing radiation, leading to changes in gene activity and expression [5]. These changes can result in variations in an individual’s phenotypic appearance with minimal impact or induce substantial structural alterations in cells, potentially leading to the development of malignant cells. Ionizing radiation is recognized as a proven epigenetic modulator.

A diet deficient in folates can pose a risk of DNA damage comparable to the changes induced by ionizing radiation. Folates play a crucial role in preventing uracil incorporation into DNA and DNA hypomethylation [32]. This function can be compromised when vitamin B12 levels are low, as it leads to a reduction in methionine synthase activity. Consequently, the concentration of S-adenosylmethionine (SAM) decreases, negatively impacting DNA methylation and hindering the conversion of dUMP to dTMP due to folate unavailability.

Both radiation exposure and folate deficiency have been linked to DNA strand breaks and alterations in the expression of various genes. Radiation activates excision processes and genes responsible for double-strand DNA repair, while mitochondrial genes are suppressed. In contrast, folate deficiency triggers base excision repair and nucleotide excision repair genes but suppresses folate-related genes. Notably, no genes involved in double-strand DNA repair are activated due to folate deficiency [29,32,33,34]. In both animal experimental models and humans, the reactivation of various herpes viruses can be induced by local trauma (e.g., surgical intervention) or systemic stress. In mice, stress leads to post-translational histone modifications (PTM) in histone tails. Viral RNA can be detected as early as two hours after stress exposure, although viral proteins are not yet encoded. Viral proteins are detected six hours after stress induction, indicating the virus’s transition out of latency. Twenty-two hours after stress, viral activity is detected in the trigeminal ganglion (TG) [40]. An example of viral reactivation is seen in the elevated body temperatures of mice to 43 °C for 10 min [40]. The appearance of HSV papules correlates with various stressors, including mental tension, fatigue, and exposure to strong light [41]. Over a century ago, it was demonstrated that trauma applied to a virus-silenced nerve, such as during chronic pain treatment, could trigger herpes outbreaks in the corresponding dermatome [42]. Other cellular stressors, such as acute protein synthesis disruption or hypoxia, are sufficient to induce viral activity. A well-documented mechanism involves the disruption of mTOR (mechanistic Target of Rapamycin) kinase activity. This enzyme plays a central role in responding to nutritional or stressful cellular events by affecting RNA translation [43].

In different tissue culture models, viral reactivation can be chemically induced in ways that affect gene activity, using compounds that, for example, block histone methylation or by making appropriate modifications to the RNK molecule [43]. Regarding HSV, external factors such as emotional stress, ionizing radiation, fever, UV exposure, hormonal changes, dental surgery, and trauma are known triggers [44]. However, it remains unclear whether these factors act directly on infected neurons or indirectly through various bodily functions. This detail is crucial in understanding the pathogenesis of latent HSV infection, as the exact mechanism of virus reactivation remains unclear and completely unpredictable [44].

One proposed mechanism involves mental stress effects mediated through virus-specific CD8 T cells. These immune cells frequently interact with infected neurons via immune synapses. CD8 T cells produce interferons, which help maintain viral latency while supporting neuronal survival [44]. Psychological and physical stressors, including ionizing radiation, influence CD8 T cell activity by releasing neuroendocrine factors. This mechanism may link HSV latency control to sympathetic nervous system activity [45].

When considering the biological process of aging, the organism itself is unable to effectively control the virus due to changes in the immune system, which has the effect of moving the latent phase of the virus towards faster reactivation. Age-related immune changes lead to increased viral activity, though the precise factors driving heightened herpes virus activity remain unclear. Research related to the stress of astronauts during space missions indicate that the level of immune suppression is sufficient to cause the reactivation of latent herpes viruses. This likely occurs through cellular immune suppression or oxidative stress caused by exposure to low doses of ionizing radiation [46].

### 10.2. Epigenetic Stress Induced by Ionizing Radiation and Reactivation of HSV-1 Virus

The host epigenome is a crucial factor in disease reactivation and significantly influences immune response, with ionizing radiation playing a key role in these changes [1,2]. These findings can be epigenetically linked to the effect of demethylated DNA molecules due to low doses of ionizing radiation and the reactivation of HSV-1, which is epigenetically dependent. Vitamin B12 and folic acid are responsible for DNA methylation, while ionizing radiation is an external factor that can cause DNA demethylation and potential HSV-1 reactivation [38]. This suggests that supplements influencing DNA methylation, as methyl group donors, may play a vital role in protecting the DNA epigenome, particularly in individuals professionally exposed to ionizing radiation [33].

An interesting study by Piyathilake et al. assessed the impact of vitamin B12 concentration on the risk of developing cervical cancer. They concluded that folates and vitamin B12 might play a significant role in reducing risks associated with the methylation of HPV 16 and lesion development [47]. A similar study by Lopes et al. found an inverse correlation between vitamin B12 intake and the presence of oncogenic HPV [48]. Additionally, a 2018 study by Ślebioda Z et al. discovered that recurrent aphthous stomatitis, including herpetic etiology, is strongly associated with vitamin B12 deficiency [49].

Immune control of viral reactivation is crucial. Correspondingly, studies on rabbits and mice have demonstrated that T cells are infiltrated in the sensory neuron of the eye region approximately 8 to 10 days after corneal infection and remain in place even after the cessation of viral activity, i.e., after the virus has stopped replicating [50,51].

Interestingly, a case study from 1956, which did not take into account the epigenetic nature of viral reactivation, reported that vitamin B12 supplementation significantly improved the clinical picture of herpetic keratitis [52]. Patients in this study experienced milder symptoms and an easier course of recurrent herpetic keratitis [52,53]. This leads to the hypothesis that viral reactivation from latency to active HSV-1 infection may depend on even a minimal deficiency of vitamin B12 or folic acid. During latency, the virus does not produce proteins, allowing it to evade the host’s immune system. However, it is unclear what maintains the connection between immune cells, such as CD8+ T-cells, and latently infected neurons.

At this point, defining the concepts of “latency” and “reactivation” is crucial, as the virus can be sporadically activated, presenting diverse clinical manifestations depending on the site of activation [38]. As previously discussed, asymptomatic viral latency may be associated with the epigenetic nature of the virus, and ionizing radiation could act as a trigger for its reactivation. Epigenetic modifications in DNA molecules are essential for human biology, as they regulate gene expression without altering the DNA’s primary structure. The epigenome modifications are partly hereditary and partly depends on the lifestyle of the individual. Epigenomic changes are greatly influenced by the external environment, including low doses of ionizing radiation, which result in alterations in gene activity, i.e., gene expression. These changes can lead to changes in the phenotypic appearance of the individual, with either minimal impact or significant structural changes in the cell, including the development of malignant cells. Food can act as a proven epigenetic modulator. Different cycles can lead to changes in the epigenome, which may be linked to deficiencies in certain dietary elements in the individual’s diet [29].

## 11. Conclusions

A minimal deficiency of vitamin B12 and folic acid can lead to changes in the epigenome that are associated with the reactivation of viruses. The presence of epigenetic changes, particularly in the model of DNA methylation, is linked to ionizing radiation. Lower reference values of vitamin B12 and folic acid, under the influence of low doses of ionizing radiation, affect the immune and epigenetic status of individuals working in ionizing radiation zones. We conclude that additional supplementation of vitamin B12 and folic acid for professionals working in ionizing radiation zones is advisable. Such supplementation provides epigenetic protection for these professionals against the stressful impact of ionizing radiation and the reactivation of potentially pathogenic viruses.

## Data Availability

No new data were created or analyzed in this study. Data sharing is not applicable to this article.
